# A Competition between Care Teams Improved Recording of Diagnoses in Primary Dental Care: A Longitudinal Follow-Up Study

**DOI:** 10.1155/2017/3080957

**Published:** 2017-10-26

**Authors:** Jouko Kallio, Timo Kauppila, Lasse Suominen, Anna Maria Heikkinen

**Affiliations:** ^1^Social and Health Services of Espoo, City of Espoo, Finland; ^2^Department of General Practice and Primary Healthcare, Institute of Clinical Medicine, University of Helsinki, Helsinki, Finland; ^3^Department of Oral and Maxillofacial Diseases and Surgery, Head and Neck Center, Helsinki University Hospital, HUS, Helsinki, Finland; ^4^Department of Oral and Maxillofacial Diseases, University of Helsinki and Helsinki University Hospital, Helsinki, Finland

## Abstract

**Introduction:**

A playful competition was launched in a primary dental health care system to improve the recording of diagnoses into an electronic patient chart system and to study what diagnoses were used in primary dental care.

**Methods:**

This was a longitudinal follow-up study with public sector primary dental care practices in a Finnish city. A one-year-lasting playful competition between the dental care teams was launched and the monthly percentage of dentists' visits with recorded diagnosis before, during, and after the intervention was recorded. The assessed diagnoses were recorded with the International Classification of Diseases (ICD-10).

**Results:**

Before the competition, the level of diagnosis recordings was practically zero. At the end of this intervention, about 25% of the visits had a recorded diagnosis. Two years after the competition, this percentage was 35% without any additional measures. The most frequent diagnoses were dental caries (K02, 38.6%), other diseases of hard tissues of teeth (K03, 14.8%), and diseases of pulp and periapical tissues (K04, 11.4%).

**Conclusions:**

Commitment to the idea that recording of diagnoses was beneficial improved the recording of dental diagnoses. However, the diagnoses obtained did not accurately reflect the reputed prevalence of oral diseases in the Finnish population.

## 1. Introduction

In primary care, the recording of diagnoses is important for ensuring sufficient treatment actions and for planning activities and for managing the resources of primary care [1–4]. Recording of diagnoses makes it possible to document the types and frequency of conditions the dentists encounter, enhance communication with patients and clinicians, enable outcomes-tracking, and facilitate data sharing and different kinds of research [5]. It also supports educational aims by enhancing learning of diagnostic skills and emphasizing link between diagnosis, treatment, and patient care [5]. Electronic patient information systems allow the recording of diagnoses and allow dentists time for clinical work instead of administrative tasks [6]. Yet, despite their availability, use of those systems has been infrequent for recording diagnoses and it has not been possible to gain a clear idea about the diagnostic distribution of treated oral diseases in primary dental care.

In the primary dental care of Espoo, the basic level of the frequency of recording oral disease diagnoses was practically 0% [4]. A higher frequency of recorded diagnoses was deemed necessary for planning activities and for managing the resources of primary care. In primary care of Espoo, it was possible to increase the frequency of recording diagnoses from 55% of all visits to general practitioners to a level of 90% by using financial group bonuses for care teams [4]. The primary dental care had no resources for financial incentives. As a solution to this problem, the administration of Espoo primary dental care started a playful competition for the dental health care teams to improve recording oral diseases diagnoses.

The aim of this study was to discover whether the competition between the dental care teams increased the rate of recording oral disease diagnoses. We also wanted to explore what diagnoses were recorded in primary dental care.

## 2. Methods

### 2.1. Design and Setting

The present work is a retrospective longitudinal quasi-experimental study with a before-and-after design in the primary dental care of the second largest city of Finland. This study was performed in Espoo city, where, in 2009, there were 230,000 inhabitants (254,000 in 2012) and 21 communal dental care teams, for example, cells. The number of dentists varied from 2 to 12 per team. There was the same number of dental nurses (including dental hygienists) supporting work of dentists in these teams, too.

### 2.2. Primary Outcomes and Data Extraction

The proportion of monthly visits having a recorded diagnosis (out of all communal primary care dentists' visits in Espoo) was our main measure to study the effect of implementing group bonuses. Diagnoses were recorded by ICD-10 system (the International Classification of Diseases (ICD), http://www.who.int/classifications/icd/en/HistoryOfICD.pdf) by the dentists. To commit the staff to the change in function, a competition was announced in December 2008 by the head of primary dental care. The nature of the reward was not revealed and it was promised solely to the team where the percentage of the dentists' visit with recorded diagnosis was the highest during the year 2009. Thus, the intervention itself took place between 1 January and 31 December 2009 (12 months). The data was obtained from the electronic Effica patient chart system (Tieto LTD, Helsinki, Finland) from which the data were reliably obtainable from 1.5.2003. No ethical approval was required because this study was performed directly by computer from the patient register without identifying the patients. According to the Finnish law about register studies, no ethical approval was required (https://rekisteritutkimusen.wordpress.com/data-protection-and-permissions/). The registry keeper (the health authorities of Espoo) granted permission to carry out the study. The report generator automatically allowed following the monthly number of recorded diagnoses for each individual doctor and therefore also by each individual communal dental care cell.

The obtained data were analyzed in a quasi-experimental design, where the recording of diagnoses was compared between similar periods of time before and after the initiation of group bonuses to all persons belonging to the cells (intervention) in primary care of Espoo city. Both the absolute and proportional amounts of visits with recorded diagnoses were available from Espoo.

The competition was closed and the reward was revealed in January 2010: the winning staff of the team got tickets to one show in the Finnish National Theatre in Helsinki with refreshments in February 2010. We continued to collect the data during 2010–2012 after the cessation of the intervention. To study what diagnoses primary care dentists used, all diagnoses were recorded during the last year of the follow-up (2012).

### 2.3. Statistical Methods

The rate of change in diagnosis marking in Espoo primary care was analyzed with a regression analysis followed by *t*-test (GLM procedure of SigmaPlot 10.0 Statistical Software, Systat Software Inc., Richmond, CA, USA) and these rates were compared with *t*-test [4]. The monthly frequencies of marking the diagnoses were analyzed by calculating monthly percentages of visits with recorded diagnoses during each of the follow-up years and comparing them with each other. The statistical significance was tested with One-Way Repeated-Measures Analysis of Variance followed by Bonferroni correction for multiple comparisons. *p* < 0.05 was considered to be statistically significant [4].

## 3. Results

**Figure 1:**
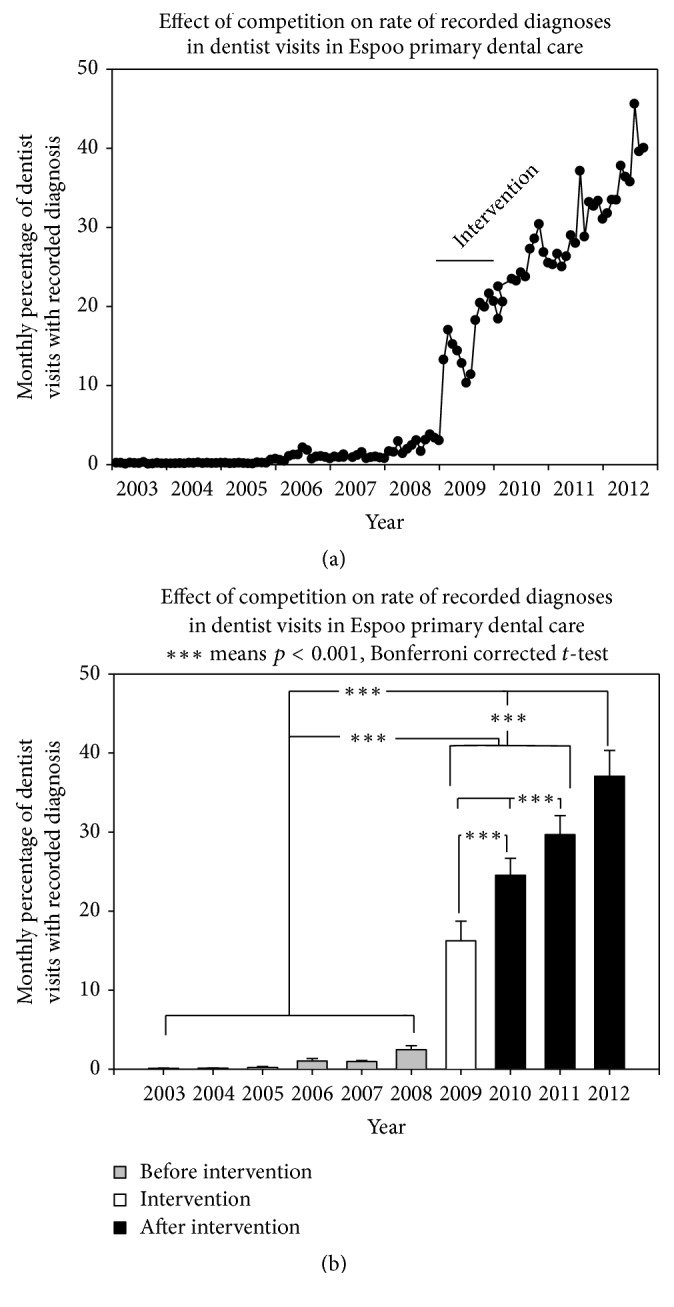
(a) Monthly percentage of dentists' visits with recorded diagnoses before, during, and after the playful competition in 2009. (b) Year-based percentage of monthly dentists' visits with recorded diagnoses during the same observation periods. Means (bars) and SDs (brackets) are presented.

The rate of change in the recording of diagnoses was 0.040 ± 0.003%/month (mean ± SEM) in 2003–2008, for example, before the intervention. During the intervention (2009), this rate increased to 0.60 ± 0.30%/month (*p* < 0.001, *t*-test). After cessation of the intervention, for example, in 2010–2012, the increased rate of recording diagnoses (0.58 ± 0.18%/month) did not differ from the rate during intervention. However, this rate was statistically significantly higher than during the preintervention level (*p* < 0.001, Figure 1(a)). Practically, this led to a situation where the monthly percentage of dentist visits having a recorded diagnosis increased every year after intervention. Once started, this increase continued every year even without any specific intervention (One-Way Repeated-Measures ANOVA, *p* < 0.001, Figure 1(b)).

According to the reported distribution of diagnoses (Table 1), the most common diagnosis assessed by the dentists was dental caries (K02). The next frequent diagnoses were other diseases of hard tissues of teeth (K03), diseases of pulp and periapical tissues (K04), and periodontal diseases (K05).

## 4. Discussion

A playful competition enhanced the marking of diagnoses significantly. Financial incentives are useful in altering the behavior of primary care dentists [7]. However, in this case, practically no financial incentives were required because the financial value of a theatre evening to one team was really a minimal investment. Furthermore, the rate of recording diagnosis in primary dental care increased after intervention, although no extra attempt to enhance this activity was performed. Thus, commitment to the idea that the recording of diagnoses was beneficial had the effect of improving the recording of diagnoses, not the competition itself. In this case, the competition just guided the attention of the primary care dentists to a significant problem which was shortage of knowledge about the quality of local clinical activities. The dentists just decided that recording diagnoses was worth doing disregarding whether they were rewarded for it or not. This led to observed improvements even after cessation of the intervention, although ICD-10 standardized oral disease diagnostic terms have not obtained a widespread extent [8, 9]. The present result is in line with former studies demonstrating that commitment of the staff is equally or even more important than financial demands when improving the quality of clinical work [10].

To the best of our knowledge, there are no former reports about the diagnoses recorded by primary care dentists. About half of the diagnostic terms entered to electronic patient chart considered dental caries, other diseases of hard tissues of teeth, or diseases of pulp and periapical tissue. The observed diagnostic terms did not accurately reflect the reputed prevalence of oral diseases in the Finnish population. According to epidemiologic surveys, periodontitis is a major common oral disease among adults in Finland and over 60% of Finnish population suffers from it [11]. However, only less than 10% of ICD-10 terms included codes related to gingivitis or periodontitis in the present study. Either communal dentists fail to diagnose these diseases, they do not record these diagnoses despite the fact that they observe their presence, or they do not record them under correct terms. Reason for this discrepancy requires further studies.

There are former studies suggesting that factors related to use of the applied diagnostic terminology itself, use of the electronic patient chart interface, or use of the terminology as part of clinic workflow may modify the frequency to record diagnoses and the quality of these recordings in dental care [12, 13]. There may also be aspects such as cultural traditions (instead of recording diagnoses, dentists are used to record treatments and procedures), extra work required to learn to use novel, possibly changing terminology, financial incentives, and fear of loss of autonomy which may decrease enthusiasm to record diagnoses [9, 14]. There is variance in how diagnostic terms are set up for use in electronic patent chart systems, and if the terms are not easy to be navigated in the category/subcategory, they may end up in the wrong place in the ICD-10 system [14]. This might be a reason why ICD-10 terms are not used in dental practice so frequently. In line with other previous findings, just ICD-10 system has been shown to be prone to misclassification bias in dental practice [15]. Yet, systems using other dental diagnostic terms are not totally protected against this type of bias either [16]. Improving electronic patient charts in accordance with existing systems of dental diagnostic terms could also enhance better recording of diagnosis in primary dental care.

The frequent use of diagnostic terms of oral disease by dentists should provide valuable data for management and for targeting proper treatments given to oral diseases and help primary dental health care to be more effective [17]. Recording diagnosis might promote diagnostic thinking and thereby enhance rational judgement of treatment options which then may lead to better treatment outcomes and increased patient safety [18]. It might also facilitate use of computer-based clinical decision support systems [18]. Habitual recording of a structured dental diagnosis would allow for the aggregation and secondary analyses of clinical data to support downstream analyses for quality improvement and epidemiological assessments and give basis for reasonable incentive systems [16]. It could also support formation of group practices, which is a current trend in dentistry [13]. As already stated in Introduction, frequent recording of diagnosis supports also educational functions in various ways [5].

## 5. Conclusion

The present data suggest that even a playful competition may be an effective primer in interventions of primary dental care. Commitment to the idea that the recording of diagnoses was beneficial had the effect of improving the recording of diagnoses. However, the quality of obtained diagnoses did not reflect well the prospected distribution of oral diseases in Finnish primary dental care.

## Figures and Tables

**Table 1:** Diagnoses made by primary care dentists.

%	Diagnosis (ICD-10 group)
2.1	Disorders of tooth development and eruption (K00-K01)

38.6	Dental caries (K02)

14.8	Other diseases of hard tissues of teeth (K03)

11.4	Diseases of pulp and periapical tissues (K04)

9.6	Gingivitis and periodontal diseases (K05)

0.2	Other disorders of gingiva and edentulous alveolar ridge (K06)

2.4	Dentofacial anomalies [including malocclusion] (K07)

1.0	Other disorders of teeth and supporting structures (K08)

0.3	Other diseases of jaws (K10)

0.1	Diseases of salivary glands (K11)

0.1	Stomatitis and related lesions (K12)

0.1	Other diseases of lip and oral mucosa (K13)

7.9	Fracture of tooth (S02)

0.3	Dislocation of tooth (S03)

10.8	Others
	*Includes:*
	*5.7%, dental examination (Z01.2)*
	*3.3%, a patient with former gastrointestinal disease (Z87.1)*
	*0.8%, bruxism (F45.8)*

## Abbreviations

ICD: The International Classification of Diseases.
